# Elucidating the Mechanism of Jisheng Shenqi Pills in the Treatment of Diabetic Kidney Disease: Network Pharmacology Combined with Experimental Verification

**DOI:** 10.2174/0118715303339433240805045749

**Published:** 2024-08-09

**Authors:** Xiaoshu Ma, Guangju Zhou

**Affiliations:** 1 College of Clinical Medicine, North Sichuan Medical College, Nanchong, China;; 2 Department of Endocrinology, Affiliated Hospital of North Sichuan Medical College, Nanchong, China

**Keywords:** Diabetic kidney disease, network pharmacology, traditional chinese medicine, AGE-RAGE axis, PI3K/AKT signaling pathway, apoptosis

## Abstract

**Background:**

While the annual incidence of diabetic kidney disease (DKD) has been soaring, the exact mechanisms underlying its onset and progression remain partially understood.

**Objective:**

The present study delved into the underlying mechanisms of Jisheng Shenqi Pill (JSP) in the treatment of DKD.

**Methods:**

The active constituents and prospective targets of JSP were identified from the Traditional Chinese Medicine Systems Pharmacology Database and Analysis Platform (TCMSP), while DKD-associated disease targets were obtained from the GeneCards database. Subsequently, Gene Ontology (GO) functional annotation and Kyoto Encyclopedia of Genes and Genomes (KEGG) pathway enrichment analyses were performed to assess the overlapping segment of drugs and disease targets. Meanwhile, a component-target-pathway network was constructed to identify pivotal components, targets, and pathways. Molecular docking and molecular dynamics simulation were also carried out to validate the binding efficacy of the pivotal components with the targets. Finally, animal experiments were conducted to corroborate the efficacy of the aforementioned targets and pathways.

**Results:**

According to bioinformatics analysis, the primary targets included JUN, TNF, and BAX, while the pivotal pathways involved were AGE/RAGE and PI3K/AKT signaling cascades. *In vivo* experiments demonstrated that JSP effectively mitigated renal impairment in DKD by reducing renal inflammation and apoptosis. This effect was presumably achieved by modulating the AGE-RAGE axis and the PI3K/AKT signaling pathway.

**Conclusion:**

Our findings imply that JSP could ameliorate renal inflammation and apoptosis in DKD mice by modulating the AGE/RAGE axis and the PI3K/AKT signaling pathway. These findings provide valuable insights into traditional Chinese medicine-based treatments for DKD.

## 1 INTRODUCTION

According to the latest edition of the International Diabetes Federation (IDF) Diabetes Atlas Report, about 537 million adults were diagnosed with diabetes worldwide in 2021, which is projected to rise to 783 million by 2045 [1]. Diabetic kidney disease (DKD) is a prevalent microvascular complication of diabetes, affecting approximately 40% of individuals with type 2 diabetes [2, 3]. It is recognized as the primary cause of end-stage renal disease (ESRD) globally [4, 5], significantly contributing to mortality rates and imposing substantial social and economic burdens on patients. The occurrence of DKD is driven by multiple factors, mainly including metabolic disorders, changes in hemodynamics, *etc*., such as the accumulation of advanced glycation end products (AGEs) in a high-sugar environment and the activation of the renin-angiotensin-aldosterone system (RAAS) [6-9]. The histopathological manifestations of DKD mainly include mesangial matrix expansion, thickening of the glomerular basement membrane (GBM), nodular lesion formation [10-12], and eventual progression to renal fibrosis. Current therapeutic strategies for DKD focus on managing blood glucose, blood pressure, and blood lipid levels alongside lifestyle modifications to enhance overall health [13, 14]. Although angiotensin-converting enzyme inhibitors and angiotensin receptor blockers are commonly prescribed in clinical practice to reduce proteinuria [15, 16], prevention of further deterioration of renal function remains challenging. Given the current scenario, exploring novel and efficacious therapeutic modalities for DKD is imperative.

Inflammation and apoptosis play critical roles in the onset and progression of DKD [17, 18]. In a hyperglycemic environment, various cytokines and chemokines are released, such as IL-1β, TNF-α, and MCP-1, which further activate caspases and induce apoptosis, exacerbating kidney damage [19-21]. Numerous reports document the presence of inflammation and apoptosis in the kidneys affected by DKD [22, 23]. However, the molecular links between inflammation, apoptosis, and DKD remain to be further elucidated.

Traditional Chinese medicine (TCM) has a longstanding history in disease management. Jisheng Shenqi Pill (JSP) - derived from prescriptions dating back to the Song Dynasty - is renowned for its therapeutic effects in warming and tonifying kidney Yang, as well as inducing diuresis to alleviate edema. JSP contains 10 traditional Chinese medicinal ingredients, including Rehmanniae Radix, Corni Fructus, Moutan Cortex, Dioscoreae Rhizoma, Poria, Alismatis Rhizoma, Cinnamomi Cortex, Aconiti Lateralis Radix Praeparata, Cyathulae Radix, and Plantaginis Semen, and exemplifies a rich pharmacological heritage in treating various ailments. Catalpol and loganin, prominent constituents of Rehmanniae Radix and Corni Fructus, respectively, exhibit hypoglycemic and anti-apoptotic properties [24]. Poricoic acid A - the primary compound in Poria - exerts an anti-fibrotic effect by mitigating renal fibrosis, thereby impeding the decline of renal function by attenuating inflammation and suppressing abnormal remodeling of the extracellular matrix (ECM) [25, 26]. Cyathulae Radix exhibits anti-inflammatory properties and can suppress apoptosis in murine renal cells [27]. Additionally, compounds such as Paeonol in Moutan Cortex, Alismatis Rhizoma polysaccharide in Alismatis Rhizoma, Cinnamaldehyde and Cinnamic Acid in Cinnamomi Cortex, *etc*., also contribute to the treatment of diabetes to a certain extent. Given the multifaceted therapeutic attributes of TCM, such as its effects on multiple components, targets, and pathways, elucidating the potential mechanism underlying JSP in the treatment of DKD is of great significance.

Network pharmacology employs computational and experimental techniques to analyze the targets of TCM and its compounds. It evaluates the regulatory impact of drugs on the biomolecular network, considering disease syndromes from a systemic and holistic standpoint. This approach is systematic, relevant, and offers predictability in understanding drug effects [28-30]. Molecular docking is a sophisticated computer-based technique that enables the prediction of interactions between ligands and targets at a molecular scale, leveraging structural information [31, 32]. Herein, we performed a network pharmacology analysis to dissect the constituents of JSP and the targets relevant to DKD to elucidate the precise mechanisms underlying the therapeutic effects of JSP in DKD. Protein-protein interaction (PPI) analysis was employed to identify pivotal target proteins. Moreover, Gene Ontology (GO) functional annotation and Kyoto Encyclopedia of Genes and Genomes (KEGG) pathway enrichment analyses were conducted to evaluate the overlapping segment of drugs and disease targets. The binding affinity of essential compounds and targets was confirmed through molecular docking, followed by molecular dynamics (MD) simulation to assess the binding stability and suitability of active compounds with therapeutic targets. Finally, the efficacy of relevant pathways and targets was confirmed through *in vivo* experiments.

## 2 MATERIALS AND METHODS

### 2.1 Screening of Bioactive Constituents and JSP Targets

Ten chemical constituents of TCM were extracted *via* the Traditional Chinese Medicine Systems Pharmacology Database and Analysis Platform (TCMSP, http://tcmspw.com/tcmsp.php). The components were screened with the criteria of oral bioavailability (OB) ≥ 30% and drug-likeness (DL) ≥ 0.18. In cases where certain chemical compositions were only partially effective or not covered by TCMSP, relevant literature was reviewed to supplement the dataset. In addition, potential targets of the effective chemical components identified in TCMSP, but lacking targets, were supplemented using The Encyclopedia of Traditional Chinese Medicine (ETCM, http://www.tcmip.cn/ETCM/).

### 2.2 Screening of DKD-associated Targets

The GeneCards database (http://www.genecards.org/) was utilized to procure relevant disease targets, employing “Diabetic Kidney diseases” and “Diabetic nephropathy” as search keywords [33]. DrugBank (https://go.drugbank.com), Therapeutic Target Database (TTD: https://db.idrblab.net/ttd/), and the Online Mendelian Inheritance in Man (OMIM) database (https://omim.org/) were referenced for supplementary information [34-36]. After eliminating duplicate entries across the four databases, DKD-associated targets were ultimately compiled.

### 2.3 Building the PPI Network

The overlapping targets between drugs and disease-related targets were acquired and depicted using JVENN [37]. The overlapping targets were then inputted into the Search Tool for the Retrieval of Interacting Genes/Proteins version 12.0 (STRING 12.0) database (https://cn.string-db.org/) to establish the PPI network [38]. Subsequently, the PPI network was visualized through Cytoscape 3.9.1 software [39], where the nodes represented the active ingredients and targets, and the edges represented the molecular interactions between them. Finally, the central targets within the PPI network were determined using the CytoNCA plugin and sorted based on their betweenness centrality [40].

### 2.4 GO and KEGG Pathway Enrichment Analyses

The Metascape database (http://metascape.org/gp/index.html#/main/step1) was employed for GO and KEGG pathway enrichment analyses of the intersection genes to uncover the mechanism underlying the efficacy of JSP in treating DKD [41]. GO enrichment analysis encompassed biological processes (BPs), molecular functions (MFs), and cellular components (CCs), subsequently sorted based on their p-values. The top 20 ranked outcomes were graphically represented using https://www.bioinformatics.com.cn, an online platform for data analysis and visualization.

### 2.5 Establishment of a Component-target-pathway (C-T-P) Network and Molecular Docking

The active components of JSP, shared targets, and KEGG pathways were inputted into Cytoscape to generate the C-T-P network. The Degree value was computed using the CytoNCA plugin, and the most highly ranked drug components were chosen for molecular docking studies with target proteins. The protein data bank (PDB) format of all target proteins was obtained from the Research Collaboratory for Structural Bioinformatics Protein Data Bank (RSCB PDB) database (https://www.rcsb.org/) and then converted into PDBQT files. Subsequently, PDBQT files containing active ingredients and target proteins were imported into AutoDockTools Vina software [42] for molecular docking to determine the minimum binding free energy between the target protein and the ligand.

### 2.6 MD Simulation

The GROMACS 2020.3 software package was employed for MD simulations of the receptor protein with the lowest binding energy to the ligand molecule identified in molecular docking results [43]. This process allowed us to ascertain the binding mode and binding energy of the protein and the ligand molecule. The initial structure obtained from molecular docking was subjected to successive energy minimization steps, including 10,000 iterations of the maximum descent method followed by 10,000 iterations of the conjugate gradient method. Subsequently, the system was equilibrated using canonical ensembles (NVT) and isothermal-isobaric ensembles (NPT) for 2000 ps, followed by 30-nanosecond MD simulations under room temperature and atmospheric pressure conditions.

## 3 EXPERIMENTAL

### 3.1 Establishment Of Animal Model and Design of Experimental Study

Sixty male C57BL/6 mice, aged 8 weeks and weighing 20 ± 2 g, were obtained from Spefu (Beijing) Biotechnology Co., LTD. The mice were housed in a specific pathogen-free (SPF) environment with controlled conditions, including temperature (24 ± 2°), humidity (50 ± 10%), and a 12-hour light and 12-hour dark cycle, and allowed free access to food and water. Following a 7-day acclimatization period, use a random number table method to randomly assign 10 mice to the normal control group and fed them on a standard diet. The remaining 30 mice were fed on a high-fat diet (HFD, comprising 60% kcal from fat, D12492 Research Diets, obtained from XiaoShuYouTai (Beijing) Biotechnology Co., Ltd) for 4 weeks. After a 12-hour fasting period, 30 mice on an HFD were intraperitoneally injected with streptozotocin (STZ, 55 mg/kg, Solarbio, Beijing, China) dissolved in 0.1 mmol L-1 pH 4.5 citrate-sodium citrate buffer for five consecutive days. Mice in the normal control group received an equivalent dose of sodium citrate buffer *via* intraperitoneal injection. Blood glucose levels were measured using a Roche blood glucose meter (ACCU-CHEK Guide, Jiangsu, China) on the 3^rd^, 7^th^, and 10^th^ days after modeling. A continuous blood glucose level ≥16.7mmol/L was considered indicative of successful model induction [44]. During the modeling process, three mice died. Additionally, eight mice had blood glucose levels below 16.7 mmol/L, leading to modeling failure and their subsequent exclusion from the experiment. The successfully modeled mice were randomly divided into the diabetic model group (model group), the treatment group (JSP group), and the irbesartan control group (IRB group). According to the requirements of the United States Food and Drug Administration (FDA), we determined the dosages of JSP and IRB using a human-to-mouse equivalent dose conversion factor of 12.3 [45]. Mice in the JSP group received oral gavage of 3.69 g/kg once daily for 12 weeks. Those in the IRB group were administered 61.5 mg/kg *via* oral gavage once daily for 12 weeks. During the experiment, nine mice succumbed to severe illness before reaching 12 weeks after modeling. Both the normal control group and the model group of mice received an equivalent dose of normal saline once daily for 12 weeks. Body weight and fasting blood glucose (FBG) levels were measured at 0, 2, 4, 8, and 12 weeks. At the end of the 12^th^ week, 24-hour urine specimens were collected using a metabolic apparatus. After gentle centrifugation at 2000 rpm for 5 min, the supernatant was separated and stored at -80°C. Subsequently, the mice were anesthetized with 1% sodium pentobarbital, followed by a collection of blood samples *via* enucleation of the mouse eyeball. Serum samples were then centrifuged at 3000 rpm for 10 min and stored at -80°C. The bilateral kidneys of the mice were collected, and their weights were measured to determine the renal index (renal index = weight of bilateral kidneys (mg) / body weight (g)). One kidney was rapidly snap-frozen in liquid nitrogen and stored at -80°C, while the other kidney underwent overnight fixation in 4% paraformaldehyde before being embedded in paraffin. This experimental protocol was approved by the Ethics Committee of North Sichuan Medical College.

### 3.2 General Condition and Biochemical Analysis

At the end of the 12^th^ week, a comprehensive assessment of the mice's overall physiological parameters was conducted, including mental status, fur condition, kidney weight, and body weight. The 24-hour urinary protein (24 h UTP) levels were evaluated using a urine protein test kit (Jiancheng, Nanjing, China). Serum creatinine (Cr) levels were quantified using a creatinine assay kit (sarcosine oxidase-based, from Jiancheng, Nanjing, China). Total cholesterol (TC) concentrations were measured with a total cholesterol assay kit (Jiancheng, Nanjing, China). Blood triglyceride (TG) levels were quantified using a triglyceride assay kit (Jiancheng, Nanjing, China).

### 3.3 Histopathological Examination of the Kidneys

After fixation in 4% paraformaldehyde for a 24-hour period, the kidney specimens were dehydrated, embedded, sectioned, and stained with hematoxylin-eosin and Masson's reagent. The pathological changes of the kidney tissues were observed under a light microscope.

### 3.4 Real-time Quantitative PCR

Total RNA was isolated from kidney tissue utilizing RNA isolater Total RNA Extraction Reagent (Vazyme, China). The concentration of total RNA concentration was determined and then it reserve-transcribed into cDNA using a PrimeScripttm FAST RT reagent Kit with gDNA Eraser (TaKaRa, Dalian, China) following the manufacturer’s instructions. The cDNA was amplified on a real-time PCR detection system with ChamQ Universal SYBR qPCR Master Mix (Vazyme, China). The relative mRNA expression was determined by the 2-ΔΔCT method. The primer sequences for IL-1β, TNF-α, MCP-1, and AGE are shown in Table **1**.

### 3.5 Enzyme-linked Immunosorbent Assay

The serum was centrifuged at 12000 g for 10 min to achieve complete lipid separation. Subsequently, the serum fasting insulin (FINS) level was evaluated using a double-antibody sandwich ELISA technique with the Mouse INS (Insulin) ELISA Kit (Fine Test, Wuhan, China). Samples and standards were added to a pre-coated anti-INS microplate following the manufacturer’s instructions, incubated, and then washed. Subsequently, a biotin-labeled antibody was applied, followed by the addition of HRP-Streptavidin Conjugate (SABC) after plate washing. Finally, the TMB substrate was added, and the optical density value was measured at a wavelength of 450 nm. The OD values of the sample were used to construct a standard curve to determine the concentration of FINS.

### 3.6 Western Blot

Kidney tissue total protein extraction was carried out utilizing RIPA high-strength lysate (Epizyme Biotech, Shanghai, China). The protein concentration was quantified and the samples were normalized using the BCA kit (Epizyme Biotech, Shanghai, China). Subsequently, protein loading buffer was added to the samples, mixed, and denatured by heating in a metal bath at 95°C for 5 min. The proteins were transferred to PVDF membranes *via* gel electrophoresis and then blocked with a commercial blocking solution for 15 min. Next, the membranes were incubated with primary antibodies targeting RAGE (rabbit, 1:1000, Cat# YP-Ab-13666, UpingBio), PI3K (rabbit, 1:500, Cat# ET1608-70, HUABIO), p-PI3K (rabbit, 1:1000, Cat# YP-Ab-17845, UpingBio), AKT (rabbit, 1:3000, Cat# ET1609-51, HUABIO), p-AKT (rabbit, 1:2000, Cat# ET1607-73, HUABIO), BAX (rabbit, 1:5000, Cat# ER0907, HUABIO), Bcl2 (rabbit, 1:2000, Cat# ET1702-53, HUABIO), and Cleaved-Caspase-3 (rabbit, 1:1000, Cat# YP-Ab-00003, UpingBio) for 12 h at 4°C. After washing, the membranes were incubated with a Goat anti-Rabbit secondary antibody (rabbit, 1:80000, Cat# HA1001, HUABIO) for 1 hour at room temperature. After incubation, the membranes were washed three times with TBST for 10 min each and then incubated with secondary antibodies for 1 hour at room temperature. The membranes were then washed three times with TBST, after which the membranes were treated with ECL ultrasensitive chemiluminescence solution. The film was then exposed to a chemiluminescence detection system and the images of the membranes were scanned.

### 3.7 Statistical Analysis

Data analysis was performed using IBM SPSS Statistics 27.0. One-way analysis of variance (ANOVA) was utilized to compare data that showed normal distribution. A *P*<0.05 was considered statistically significant.

## 4 RESULTS

### 4.1 Identification of Active Ingredients and Targets of JSP

**Fig. (1):**
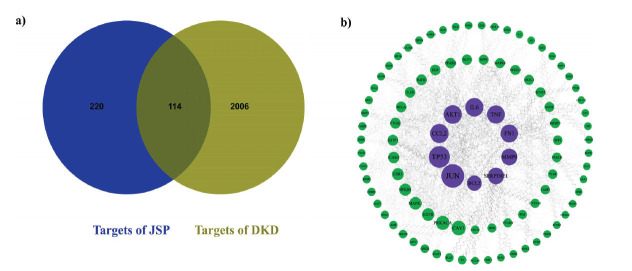
(**a**) Venn diagram of the 114 common objects. (**b**) Protein-protein interaction network. Purple represents the top 10 core targets.

Utilizing the TCMSP database as a foundation, complemented by data from the ETCM database and relevant literature, 81 active compounds and 334 predicted molecular targets were identified after eliminating duplications. The protein targets related to the aforementioned active substances were standardized through gene name conversion using the UniProt database. The GeneCards database, supplemented by data from OMIM, DrugBank, and TTD databases, was utilized to explore disease-related targets for target validation. Following the median screening, 2,120 DKD-related targets were identified. Overlapping targets between drug targets and disease-related targets were identified using JVENN. Detailed information is depicted in Fig. (**1a**). The obtained 114 intersection targets were uploaded to the STRING database to establish the PPI network. Subsequently, the obtained PPI network was imported to Cytoscape (Fig. **1b**). The top 10 core targets identified were JUN, TP53, CCL2, AKT1, IL6, TNF, FN1, MMP9, SERPINE1, and BCL2. These targets were likely the central focus of JSP in treating DKD. Notably, most of these targets were inflammation-related targets, followed by apoptosis-related targets. This suggests that JSP may ameliorate DKD by potentially impacting inflammation and cellular apoptosis pathways. Moreover, it also engages targets linked to tumorigenesis.

**Fig. (2):**
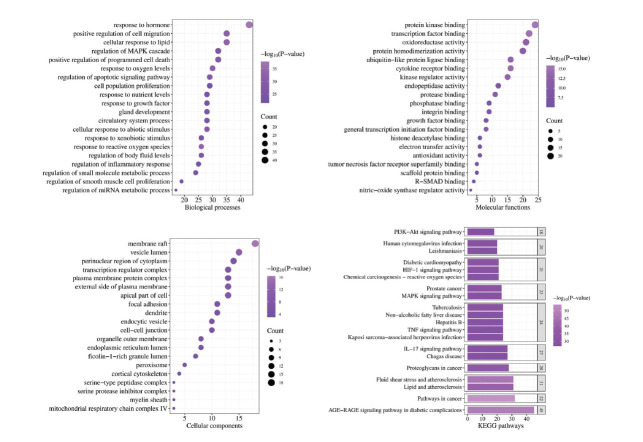
GO functional annotation and KEGG pathway enrichment analyses.

**Fig. (3):**
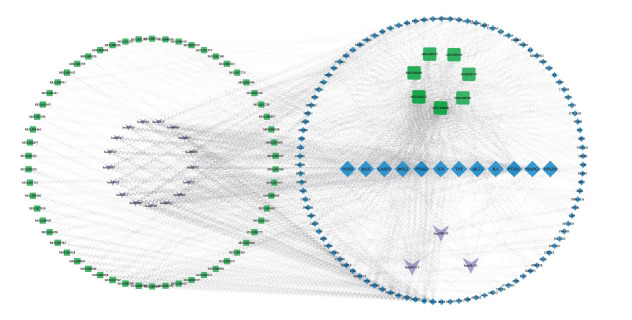
Component-target-pathway (C-T-P) network. Green represents the active ingredient, blue represents the core target, and purple represents the core pathway.

GO and KEGG pathway enrichment analyses of the 114 intersecting genes were conducted using the Metascape database (Fig. **2**). BPs were primarily related to hormone response, reactive oxygen species response, lipid response in cells, regulation of apoptotic signaling pathways, positive regulation of programmed cell death, regulation of inflammatory response, cell population proliferation, *etc*. MFs were predominantly associated with protein kinase binding, cytokine receptor binding, growth factor receptor binding, antioxidant activity, tumor necrosis factor receptor superfamily binding, nitric oxide synthase regulatory activity, *etc*. CCs were mainly linked to membrane raft, vesicle lumen, mitochondrial respiratory chain complex IV, peroxisome, *etc*. KEGG pathway enrichment analysis revealed that these genes were mainly enriched in advanced glycation end product (AGE)/receptor for AGE (RAGE) signaling pathway in diabetic complications, lipid and atherosclerosis pathways, interleukin-17 (IL-17) signaling pathway, tumor necrosis factor (TNF) signaling pathway, mitogen-activated protein kinase (MAPK) signaling pathway, phosphoinositide 3-kinase (PI3K)/protein kinase B (AKT) signaling pathway, *etc*. The active constituents of drugs, the common targets at the intersection, and the pathways identified through KEGG pathway enrichment analysis were consolidated into network and attribute files. These files were then integrated into Cytoscape to generate a C-T-P network (Fig. **3**). The centrality of the C-T-P network was computed and arranged by size, and the top-ranked core targets included PTGS2, PTGS1, AKT1, NOS3, JUN, CASP3, MAPK1, TNF, NFKB1, BAX, BCL2, and IL6. Notably, the majority of these core targets were associated with inflammation and apoptosis. This further reinforces the idea that JSP could potentially alleviate DKD by modulating these processes. Considering that a compound formulation was utilized, accounting for all drugs was crucial. Overall, the key components of JSP for the treatment of DKD were Quercetin, Kaempferol, Stigmasterol, Wogonin, Beta-sitosterol, Baicalein, and Dehydroeburicoic acid, whereas the key pathways involved were the AGE/RAGE signaling pathway, PI3K/AKT signaling pathway, MAPK signaling pathway, *etc*.

**Fig. (4):**
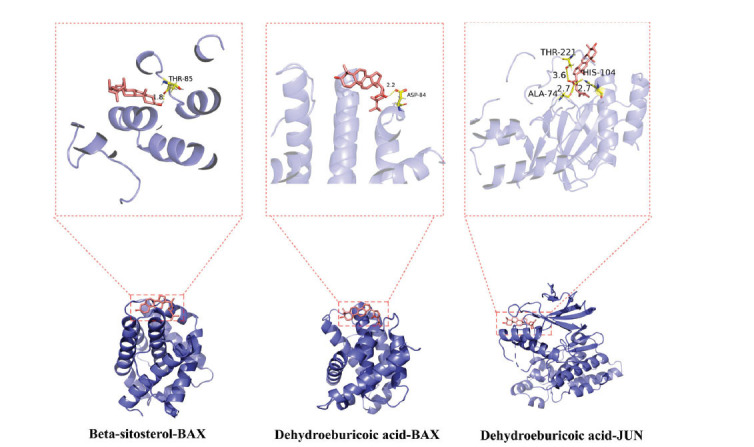
Visualization of the top three best results in terms of docking energy among molecular docking results.

Top 10 core targets associated with inflammation and apoptosis were chosen for molecular docking with the aforementioned key compounds. It is widely acknowledged that the affinity between a protein and a ligand can be quantified through the binding energy during docking. Generally, a lower binding energy indicates a more stable and probable interaction. Binding energies below -5 Kcal/mol were considered robust based on prior studies [16]. According to the docking outcomes, TNF, BAX, and JUN exhibited the most favorable binding affinity and strongest binding effect when paired with the active components of the drug (Table **2**). The three most energetically favorable binding configurations were Beta-sitosterol-BAX, Dehydroeburicoic acid-BAX, and Dehydroeburicoic acid-JUN, and were visualized using PyMOL (The PyMOL Molecular Graphics System, http://www.pymol.org.) (Fig. **4**). Subsequently, MD simulations were conducted for 30 nanoseconds on these three optimal binding configurations, exhibiting binding free energies of -18.646 kcal/mol, -24.317 kcal/mol, and -48.088 kcal/mol, respectively. Root mean square deviation (RMSD), root mean square fluctuation (RMSF), and radius of gyration (Rg) curves portray the variability in protein conformation, the fluctuation in amino acid residues within the protein, and the overall compactness of the protein structure, respectively [46]. As shown in Fig. (**5**), RMSD curves for all three protein complexes exhibit stability, suggesting minimal structural changes following ligand binding. Conversely, RMSF curves reveal high flexibility in amino acid residues. Rg curves demonstrate a stable gyration radius across the three protein complexes. Collectively, these results further confirm the good stability of the protein-ligand binding interaction.

### 4.2 Therapeutic Effect of JSP on DKD Mice

**Fig. (6):**
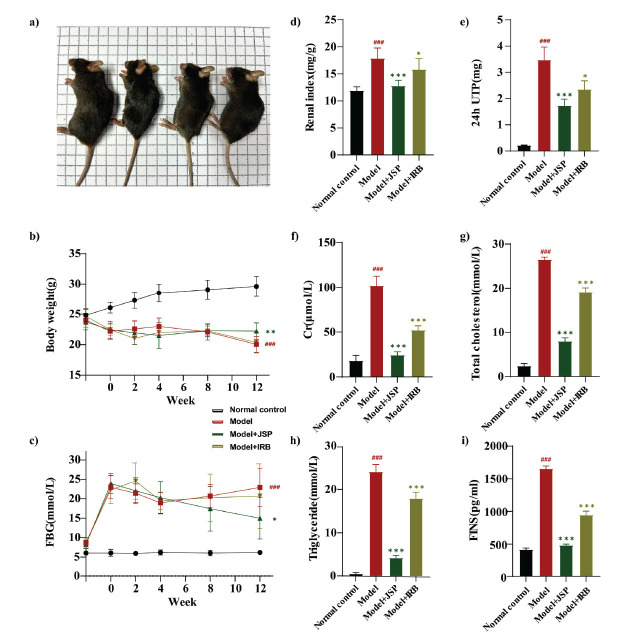
(**a**) Photos of mice, from left to right, Normal control, Model, Model+IRB and Model+JSP. After 12 weeks of treatment, changes in body weight (**b**), FBG (**c**), and renal index (**d**) were observed in each group of mice. The changes in biochemical indexes such as UTP (**e**), Cr (**f**), TC (**g**), TG (**h**) and FINS (**i**) were evaluated. Data are presented as mean ± standard deviation. ^###^*p* < 0.001 means compared with the normal control group; ***p <0.001 indicates that compared with the model group; **p <0.01 indicates that compared with the model group; *p <0.05 indicates comparison with the model group.

**Fig. (7):**
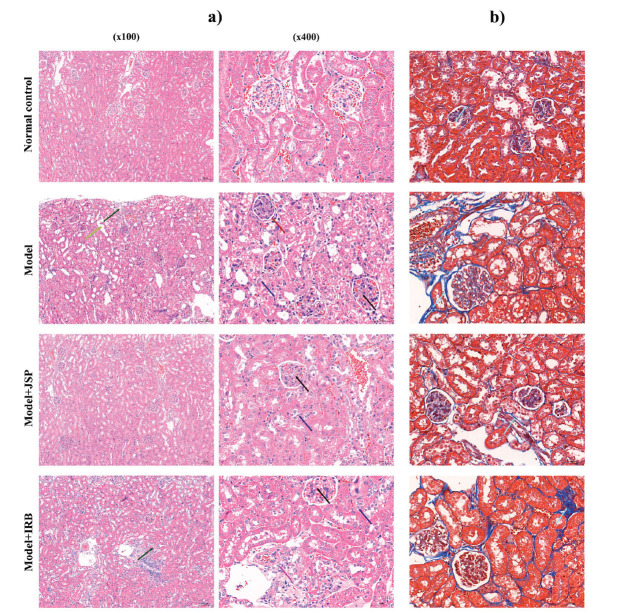
HE staining (**a**) and Masson staining (**b**) of renal tissue. Dark green arrows represent degeneration and necrosis of renal tubular epithelial cells. The light green arrow represents the dilatation of the renal tubular lumen. Red arrows represent glomerular basement membrane thickening; Dark blue arrows represent degeneration of renal tubular epithelial cells; Black arrows represent mesangial proliferation; Purple arrows represent necrotic cell debris; Light blue arrows represent fibrous expression.

After a 12-week intervention period, mice in the model group displayed diminished mental states and body sizes and greasier fur compared with those in the normal group. However, JSP-treated mice exhibited improved conditions compared with the model group. The comparison between mice in each group is depicted in Fig. (**6a**). Furthermore, marked differences in body weight (BW), fasting blood glucose (FBG), and renal index (RI) were observed between the two mouse groups (Figs. **6b**-**d**). The mice in the model group exhibited a substantial decrease in BW and significant increases in FBG and RI compared with the control group. JSP treatment significantly elevated BW (*p* <0.01) and reduced FBG (*p* <0.05) and RI (*p* <0.001). However, no substantial changes in BW and FBG were observed among irbesartan (IRB)-treated mice compared with the model group, despite a significant decrease in RI (*p* <0.05). Compared with the normal control group, the model group demonstrated substantial elevations in 24-hour urine total protein (UTP), creatinine (Cr), total cholesterol (TC), and triglyceride (TG) levels (Figs. **6e**-**h**). Nonetheless, JSP intervention resulted in dramatic reductions in 24-hour UTP, Cr, TC, and TG (all *p* <0.001), underscoring superior treatment efficacy compared with IRB. Compared with the normal control group, fasting insulin (FINS) levels were significantly elevated in the model group (Fig. **6i**). However, JSP treatment significantly reduced FINS levels (*p* <0.001), with the treatment effect significantly surpassing that of IRB. Hematoxylin and eosin staining revealed that mice in the model group displayed marked mesangial proliferation, thickening of the basement membrane, and degeneration, necrosis, and dilation of renal tubular epithelial cells, all of which were significantly different from those observed in the control group. Additionally, Masson’s trichrome staining demonstrated prominent renal fibrosis in model group mice. Conversely, treatment with JSP and IRB improved renal condition, with the efficacy of JSP being superior to that of IRB (Fig. **7**).

### 4.3 Effects of JSP on the AGE-RAGE Axis, PI3K/AKT Pathway, Renal Inflammation, and Cellular Apoptosis in DKD Mice

**Fig. (8):**
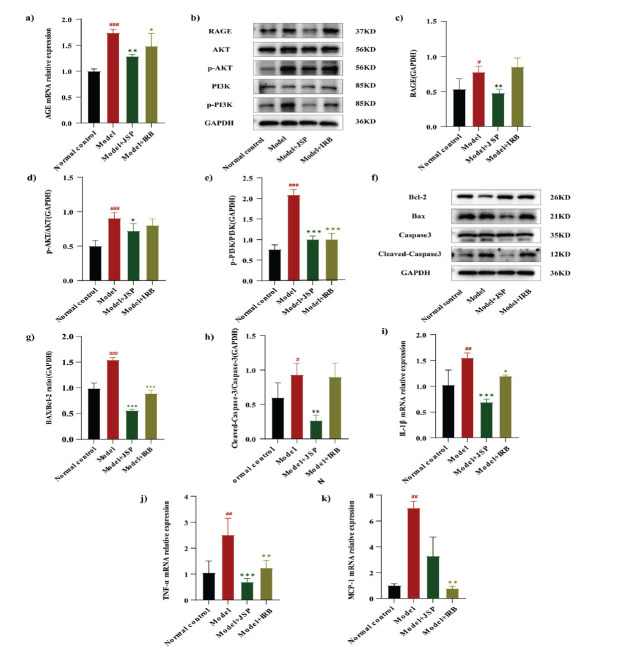
(**a-e**) JSP treatment improved the mRNA expression of AGE and the protein expression levels of RAGE, p-AKT/AKT and p-PI3K/PI3K in the kidney of DKD mice. (**f-h**) JSP treatment could improve the expression levels of BAX/Bcl-2 ratio, Cleaved-Caspase-3 and Bcl2 proteins in the kidney of DKD mice. (**i-k**) JSP treatment could improve the mRNA expression of IL-1β, TNF-α and MCP-1 in DKD mice kidney. ^###^*p* < 0.001 means compared with the normal control group; ^##^*p* <0.01 means compared with the normal control group; ^#^*p* <0.05 indicates comparison with the normal control group; ***p <0.001 indicates that compared with the model group; **p <0.01 indicates that compared with the model group; *p <0.05 indicates comparison with the model group.

The real-time quantitative PCR (RT-qPCR) findings revealed that mRNA expression levels of AGE were substantially elevated in the model group compared with the normal control group (*p* <0.001). Nevertheless, these levels were significantly decreased after JSP treatment (*p* <0.01), with JSP demonstrating a notably superior treatment effect to IRB (Fig. **8a**). Western blot analysis revealed that protein expression levels of RAGE (*p* <0.05), p-AKT/AKT (*p* <0.001), and p-PI3K/PI3K (*p* <0.001) were significantly higher in the model group than in the normal control group. However, an opposite trend was observed after JSP treatment (Figs. **8b**-**e**). Taken together, these data indicate that JSP can inhibit the AGE-RAGE axis and suppress the PI3K/AKT pathway.

Furthermore, the protein expression levels of Cleaved-Caspase (CASP)-3 were significantly increased in the kidney tissue of the model group compared with the normal control group (*p* <0.05), whereas the BAX/Bcl-2 ratio was also significantly increased (*p* <0.001), indicating increased apoptosis in the kidney tissue of the model group mice. The BAX/Bcl-2 ratio significantly differed between the IRB-treated group and the model group (*p* <0.001), whereas protein expression levels of Cleaved-CASP-3 showed no significant difference between the two groups (*p* >0.05). Meanwhile, the BAX/Bcl-2 ratio (*p* <0.001) and the protein levels of Cleaved-CASP-3 (*p* <0.01) were significantly reduced in the JSP-treated group compared with the model group, indicating a decrease in renal cellular apoptosis after JSP treatment, which demonstrated superior efficacy to the IRB treatment (Figs. **8f**-**h**).

Compared with the normal control group, the model group exhibited increased mRNA expression of IL-1β, TNF-α, and monocyte chemoattractant protein-1 (MCP-1) in renal tissue, indicating pronounced inflammation in the kidneys of mice in the model group. However, JSP treatment significantly reduced the mRNA expression of IL-1β (*p* <0.001) TNF-α (*p* <0.001). However, although the mRNA expression of MCP-1 was decreased, the difference was not significant. Furthermore, treatment with IRB significantly decreased the mRNA expression levels of IL-1β (*p* <0.05), TNF-α (*p* <0.01), and MCP-1 (*p* <0.01) (Figs. **8i**-**k**). Altogether, these findings suggest that both JSP and IRB possess therapeutic efficacy in managing renal inflammation, with IRB demonstrating superior effectiveness in controlling inflammation compared to JSP.

## 5 DISCUSSION

The annual prevalence of DKD is rising steadily, prompting an increasing need for novel treatment modalities. Han *et al*. [47] found that Yi-Shen-Hua-Shi granule ameliorated DKD *via* the ‘gut-kidney axis’ pathway. Liu *et al*. [48] demonstrated that ShenKang injection mitigated AGEs-induced oxidative stress damage in HK-2 cells and enhanced DKD by activating the Keap1/Nrf2/Ho-1 signaling pathway. Ji *et al*. [49] noted that emodin conferred renal protective benefits on DKD by suppressing ferroptosis and reinstating Nrf2-mediated antioxidant capacity. At present, considerable attention is focused on traditional herbal medicine for DKD treatment. Hence, the present study utilized bioinformatics analysis in conjunction with *in vivo* experimental validation to investigate the mechanism of action of JSP in treating DKD.

Bioinformatics analysis of the pivotal targets associated with the efficacy of JSP in treating DKD was performed, key constituents were identified, such as Quercetin, Kaempferol, Stigmasterol, Wogonin, Beta-sitosterol, Baicalein, and Dehydroeburicoic acid. The core targets encompassed PTGS2, PTGS1, AKT1, NOS3, JUN, CASP3, MAPK1, TNF, NFKB1, BCL2, and BAX. The essential pathways implicated included the AGE-RAGE signaling pathway, PI3K-AKT signaling pathway, and MAPK signaling pathway. The most effective compound-target interactions were Beta-sitosterol-BAX, Dehydroeburicoic acid-BAX, and Dehydroeburicoic acid-JUN. A certain dosage of Quercetin was previously to enhance the expression of pro-apoptotic proteins while decreasing the levels of anti-apoptotic proteins, exhibiting anti-inflammatory properties [50]. Kaempferol can modulate the M1/M2 polarization of glomerular macrophages, leading to reduced levels of TNF-α and IL-1β [51]. Additionally, it upregulates the anti-apoptotic protein BCL-2 and downregulates pro-apoptotic proteins BAX and CASP-3 in db/db mice [52], thus ameliorating renal inflammation and cellular apoptosis in DKD mice. Stigmasterol can mitigate the accumulation of free cholesterol and reactive oxygen species (ROS), shielding pancreatic β-cells against glucolipotoxicity by augmenting insulin content and diminishing early apoptosis markers [53]. Wogonin can modulate autophagy and inflammation through the PI3K/Akt/nuclear factor-kappa B (NF-κB) signaling pathway, thereby mitigating tubulointerstitial fibrosis [54]. Beta-sitosterol mitigates the phosphorylation of insulin receptor substrate-1 (IRS-1) induced by high fat and sucrose intake, concurrently enhancing the expression of the insulin receptor, IRS-1, AKT, and other proteins and genes [55]. Furthermore, it reduces the BAX/BCL-2 ratio and suppresses the CASP-3 cascade reaction, thus mitigating cellular apoptosis [56]. Baicalein exhibits inhibitory effects on TNF-α in renal tissues, decreases the deposition of ECM in tubular cells, mitigates renal inflammation, and hinders tubular atrophy [57]. Dehydroeburicoic acid can modulate the expression levels of p-AKT/AKT in streptozotocin (STZ) combined with high-fat diet (HFD)-induced diabetic mice, thereby mitigating systemic insulin resistance and fat accumulation [58]. Collectively, these pieces of evidence indicate that the active constituents of JSP can regulate the PI3K/AKT signaling pathway, potentially alleviating renal inflammation and cellular apoptosis in DKD mice.

Further, we induced a DKD model and administered continuous intervention for 12 weeks. Albuminuria serves as an early indicator of DKD, whereas serum Cr has traditionally been utilized as a marker for glomerular filtration [59]. Our data revealed a significant rise in urinary albumin and serum Cr levels in the model group. Individuals with diabetes are susceptible to developing hyperlipidemia - a condition that exacerbates the decline in kidney function [60]. Hence, managing aberrant blood lipid levels in diabetic individuals is imperative. Hematoxylin and eosin and Masson’s trichrome staining revealed characteristic kidney alterations associated with DKD in the model group. However, JSP treatment markedly ameliorated the aforementioned biochemical markers and renal manifestations of DKD. In addition, IRB was utilized as a positive control drug in the current study. Results showed that the therapeutic efficacy of JSP in DKD mice surpassed that of IRB to a certain extent.

Inflammation and cellular apoptosis are pivotal factors in the progression of DKD. Our bioinformatics analysis demonstrated that targeting inflammation and cellular apoptosis may serve as pivotal strategies in treating DKD with JSP. Elevated blood glucose levels trigger cellular injury, prompting the secretion of chemotactic factors such as MCP-1 and cellular adhesion molecules. These factors facilitate macrophage recruitment to the kidneys during the initial phases of DKD [9]. The recruited macrophages in DKD, predominantly the M1 subtype, generate an array of renal injury-inducing factors such as TNF-α, IL-1β, *etc* [61]. This sets off an inflammatory cascade, intensifying renal damage and fostering persistent inflammation [62]. Cell apoptosis is programmed cellular death, modulated by an equilibrium between pro-apoptotic and anti-apoptotic proteins, along with initiator caspases and effector caspases [63]. BAX is an apoptotic-inducing protein critical for mitochondrial modulation of cellular apoptosis. Elevated BAX levels facilitate the initiation of early-stage cellular apoptosis [64]. The BAX/Bcl-2 ratio is an important predictor of apoptosis [65]. The interaction between BAX and BCL-2 leads to the formation of a complex, which ultimately triggers the degradation of BCL-2 and facilitates the process of cellular apoptosis [66]. Cleaved-CASP-3 is a pivotal executor of cellular apoptosis and demonstrates pronounced efficacy in mitochondria-mediated cell apoptosis [67]. Our study indicates that JSP exhibits a marked effect in mitigating renal inflammation and cellular apoptosis in DKD mice.

Furthermore, we explored the impacts of JSP on the AGE-RAGE axis and the PI3K/AKT signaling pathway. Prolonged elevation in blood glucose levels can increase the accumulation of AGEs in diabetic individuals. AGEs contribute to the development of diabetic kidney disease (DKD). The interaction between AGEs and RAGE can activate multiple downstream pathways, leading to kidney inflammation, proliferation, and apoptosis. It can stimulate the production of TNF-α, IL-1β, TGF-β, and ZEB2, as well as other cytokines and transcription factors. The interaction between AGEs and RAGE in podocytes, mesangial epithelial cells, and renal tubular cells increases TGF-βexpression, enhancing the synthesis of extracellular matrix components such as type IV collagen, laminin, and fibronectin, leading to basement membrane thickening. The interaction also promotes the production of ZEB2, mediating the epithelial-mesenchymal transition of podocytes, leading to basement membrane detachment. These factors contribute to renal fibrosis and the production of proteinuria. In addition, it increases ROS production and oxidative stress, further exacerbating renal inflammation and fibrosis, ultimately causing structural damage and renal dysfunction [68]. PI3K serves as a pivotal regulatory element within the signaling pathway responsible for governing cell growth, metabolism, and apoptosis [69]. AKT is a critical signaling molecule positioned downstream of PI3K. AKT accumulates on the cell membrane after PI3K activation, where its activation triggers a series of biological functions, such as cell proliferation, differentiation, apoptosis, migration, and metabolism [70]. The activation of the PI3K/AKT pathway has been observed in diabetic renal tubular cells. Existing evidence indicates that silencing the negative regulator SHIP in HK2 cells results in the activation of the PI3K/AKT pathway, subsequently leading to increased expression of TGF-β1, collagen III (COL III), COL I, and fibronectin [71]. This suggests that inhibiting the PI3K/AKT pathway may hinder the accumulation of the ECM induced by high glucose and fibrosis associated with diabetic nephropathy. The present study found that JSP suppressed the activation of the AGE-RAGE axis and the PI3K/AKT pathway, leading to improvements in renal pathologies observed in DKD, such as mesangial proliferation, basement membrane thickening, tubular epithelial cell degeneration, necrosis, and the extent of renal fibrosis.

The AGE-RAGE axis can modulate numerous cellular events *via* the PI3K/AKT signaling pathway. The interaction between AGE and RAGE triggers activation of the PI3K/AKT pathway, possibly leading to increased production of inflammatory mediators, which may cause tissue and organ damage [72]. Bao *et al*. [73] demonstrated that the interplay between AGE-RAGE modulates the subsequent PI3K/AKT signaling cascade, impacting cell proliferation and apoptosis through ubiquitination pathways. Sharma *et al*. [74] showed that the interplay between AGE and RAGE enhances the phosphorylation of PI3K and AKT. Heightened AKT levels can enhance the functionality of the NF-κB protein complex, intensifying the inflammatory reaction [75], and can also modulate the BAX/BCL-2 ratio, indirectly influencing apoptosis [76].

## CONCLUSION

In summary, the current study highlights the significant efficacy of JSP in managing STZ-induced DKD in mice. Pathological staining results demonstrated that JSP can significantly ameliorate glucose and lipid metabolism in DKD mice, mitigate renal inflammation and apoptosis, and safeguard renal structure and functionality. These effects are likely achieved through the modulation of the AGE-RAGE axis, partially influencing the PI3K/AKT signaling pathway either directly or indirectly. These discoveries offer insights into the potential mechanisms underlying JSP treatment of DKD. Nevertheless, the specific mechanism by which JSP regulates the PI3K/AKT signaling pathway *via* the AGE-RAGE axis remains ambiguous, necessitating further *in vitro* experiments and alternative methodologies to elucidate and substantiate these mechanisms and identify novel therapeutic approaches for DKD.

## Figures and Tables

**Fig. (5):**
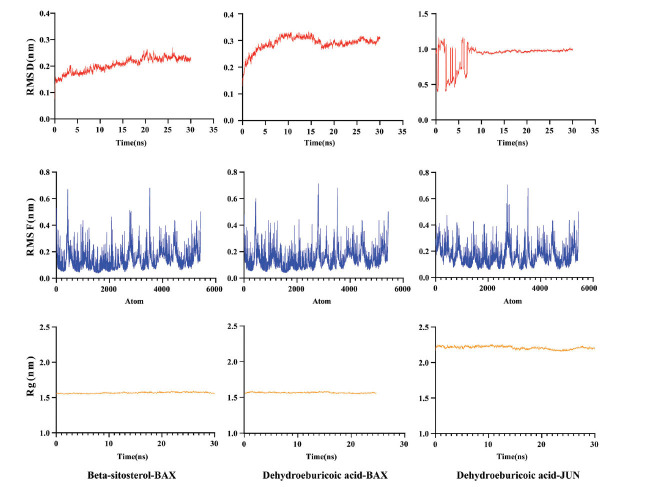
RMSD, RMSF, and Rg curves of molecular dynamics simulations are shown.

**Table 1:** Primer sequences for RT-qPCR.

**Gene**	**Sequences (5’-3’**)
GAPDH	CAGTGGCAAAGTGGAGATTGTTG (forward)
	TCGCTCCTGGAAGATGGTGAT (reverse)
AGE	TGGATGTATTGTCGCTTATACCG (forward)
	AACGCAGCAAAAATTCACCAC (reverse)
IL-1β	GAAATGCCACCTTTTGACAGTG (forward)
	TGGATGCTCTCAGGACAG (reverse)
TNF-α	CCTGTAGCCCACGTCGTAG (forward)
	GGGAGTAGACAAGGTACAACCC (reverse)
MCP-1	TAAAAACCTGGATCGGAACCAAA (forward)
	GCATTAGCTTCAGATTTACGGGT (reverse)

**Table 2:** Molecular docking partial binding energy results (Kcal/mol).

**Ligand**	**Receptor**		
	**BAX**	**TNF**	**JUN**
MOL000098	-6.01	-6.45	-6.29
FMOL000422	-6.68	-5.84	-6.33
MOL000449	-5.73	-6.83	-6.15
MOL000173	-6.87	-5.25	-6.16
MOL000358	-8.19	-6.08	-6.03
MOL002714	-6.53	-5.37	-6.14
MOL000300	-8.02	-6.15	-7.37

## Data Availability

The data that support the findings of this study are available from the corresponding author [G.Z.] upon request.

## LIST OF ABBREVIATIONS

24 h UTP: 24-hour Urinary Protein
AGEs: Advanced Glycation End Products
Cr: Creatinine
DKD: Diabetic Kidney Disease
FBG: Fasting Blood Glucose
FINS: Fasting Insulin
GO: Gene Ontology
HFD: High-fat Diet
IRB: Irbesartan
JSP: Jisheng Shenqi Pill
KEGG: Kyoto Encyclopedia of Genes and Genomes
MD: Molecular Dynamics Simulation
PPI: Protein-protein Interaction
STZ: Streptozotocin
TC: Total Cholesterol
TCMSP: Traditional Chinese Medicine Systems Pharmacology Database and Analysis Platform
TG: Triglyceride

## References

[1] Sun H., Saeedi P., Karuranga S., Pinkepank M., Ogurtsova K., Duncan B.B., Stein C., Basit A., Chan J.C.N., Mbanya J.C., Pavkov M.E., Ramachandaran A., Wild S.H., James S., Herman W.H., Zhang P., Bommer C., Kuo S., Boyko E.J., Magliano D.J. (2022). IDF Diabetes Atlas: Global, regional and country-level diabetes prevalence estimates for 2021 and projections for 2045.. Diabetes Res. Clin. Pract..

[2] Johansen K.L., Chertow G.M., Foley R.N., Gilbertson D.T., Herzog C.A., Ishani A., Israni A.K., Ku E., Kurella Tamura M., Li S., Li S., Liu J., Obrador G.T., O’Hare A.M., Peng Y., Powe N.R., Roetker N.S., St Peter W.L., Abbott K.C., Chan K.E., Schulman I.H., Snyder J., Solid C., Weinhandl E.D., Winkelmayer W.C., Wetmore J.B., US Renal Data System (2021). US renal data system 2020 annual data report: Epidemiology of kidney disease in the United States.. Am. J. Kidney Dis..

[3] Bastos R.M.C., Simplício-Filho A., Sávio-Silva C., Oliveira L.F.V., Cruz G.N.F., Sousa E.H., Noronha I.L., Mangueira C.L.P., Quaglierini-Ribeiro H., Josefi-Rocha G.R., Rangel É.B. (2022). Fecal microbiota transplant in a pre-clinical model of type 2 diabetes mellitus, obesity and diabetic kidney disease.. Int. J. Mol. Sci..

[4] Gupta S., Dominguez M., Golestaneh L. (2023). Diabetic kidney disease.. Med. Clin. North Am..

[5] Fountoglou A., Deltas C., Siomou E., Dounousi E. (2024). Genome-wide association studies reconstructing chronic kidney disease.. Nephrol. Dial. Transplant..

[6] Tian Y., Gao Z., Liu W., Li J., Jiang X., Xin Y. (2022). Unveiling the vital role of long non-coding RNAs in cardiac oxidative stress, cell death, and fibrosis in diabetic cardiomyopathy.. Antioxidants.

[7] Rustiasari U.J., Roelofs J.J. (2022). The role of platelets in diabetic kidney disease.. Int. J. Mol. Sci..

[8] Wei J., Zhao Y., Liang H., Du W., Wang L. (2022). Preliminary evidence for the presence of multiple forms of cell death in diabetes cardiomyopathy.. Acta Pharm. Sin. B.

[9] Rayego-Mateos S., Morgado-Pascual J.L., Opazo-Ríos L., Guerrero-Hue M., García-Caballero C., Vázquez-Carballo C., Mas S., Sanz A.B., Herencia C., Mezzano S., Gómez-Guerrero C., Moreno J.A., Egido J. (2020). Pathogenic pathways and therapeutic approaches targeting inflammation in diabetic nephropathy.. Int. J. Mol. Sci..

[10] Oshima M., Shimizu M., Yamanouchi M., Toyama T., Hara A., Furuichi K., Wada T. (2021). Trajectories of kidney function in diabetes: A clinicopathological update.. Nat. Rev. Nephrol..

[11] Ma L., Li J., Zhang X., Zhang W., Jiang C., Yang B., Yang H. (2024). Chinese botanical drugs targeting mitophagy to alleviate diabetic kidney disease, a comprehensive review.. Front. Pharmacol..

[12] Kourtidou C., Tziomalos K. (2023). The role of histone modifications in the pathogenesis of diabetic kidney disease.. Int. J. Mol. Sci..

[13] Forst T., Mathieu C., Giorgino F., Wheeler D.C., Papanas N., Schmieder R.E., Halabi A., Schnell O., Streckbein M., Tuttle K.R. (2022). New strategies to improve clinical outcomes for diabetic kidney disease.. BMC Med..

[14] Jarraya F., Niang A., Bagha H., Tannor E.K., Sumaili E.K., Wan D.I.M., Chothia M.Y., Mengistu Y.T., Kaze F.F., Ulasi I.I., Naicker S., Hafez M.H., Yao K.H. (2024). The role of sodium-glucose cotransporter-2 inhibitors in the treatment paradigm of CKD in Africa: An African association of nephrology panel position paper.. Kidney Int. Rep..

[15] American Diabetes Association (2020). 11. Microvascular complications and foot care: *Standards of medical care in diabetes−2020*.. Diabetes Care.

[16] Mirioglu S., Uludag O., Hurdogan O., Kumru G., Berke I., Doumas S.A., Frangou E., Gul A., Amyloidosis A.A. (2024). AA amyloidosis: A contemporary view.. Curr. Rheumatol. Rep..

[17] Wu T.H., Chang L.H., Chu C.H., Hwu C.M., Chen H.S., Lin L.Y. (2022). Soluble tumor necrosis factor receptor 2 is associated with progressive diabetic kidney disease in patients with type 2 diabetes mellitus.. PLoS One.

[18] Scurt F.G., Menne J., Brandt S., Bernhardt A., Mertens P.R., Haller H., Chatzikyrkou C. (2024). Endostatin, soluble tumour necrosis factor receptor 1 and soluble tumour necrosis factor receptor 2 cannot predict new onset of microalbuminuria in patients with type 2 diabetes.. Diabetes Metab. Res. Rev..

[19] Wu T.J., Hsieh Y.J., Lu C.W., Lee C.J., Hsu B.G. (2021). Linagliptin protects against endotoxin-induced acute kidney injury in rats by decreasing inflammatory cytokines and reactive oxygen species.. Int. J. Mol. Sci..

[20] Wu Q., Huang F. (2024). Targeting ferroptosis as a prospective therapeutic approach for diabetic nephropathy.. Ann. Med..

[21] Cai H., Zeng Y., Luo D., Shao Y., Liu M., Wu J., Gao X., Zheng J., Zhou L., Liu F. (2024). Apoptosis and NETotic cell death affect diabetic nephropathy independently: An study integrative study encompassing bioinformatics, machine learning, and experimental validation.. Genomics.

[22] Myakala K., Wang X.X., Shults N.V., Krawczyk E., Jones B.A., Yang X., Rosenberg A.Z., Ginley B., Sarder P., Brodsky L., Jang Y., Na C.H., Qi Y., Zhang X., Guha U., Wu C., Bansal S., Ma J., Cheema A., Albanese C., Hirschey M.D., Yoshida T., Kopp J.B., Panov J., Levi M. (2023). NAD metabolism modulates inflammation and mitochondria function in diabetic kidney disease.. J. Biol. Chem..

[23] Wang T., Chen Y., Liu Z., Zhou J., Li N., Shan Y., He Y. (2024). Long noncoding RNA Glis2 regulates podocyte mitochondrial dysfunction and apoptosis in diabetic nephropathy *via* sponging miR-328-5p.. J. Cell. Mol. Med..

[24] Chen Y., Chen J., Jiang M., Fu Y., Zhu Y., Jiao N., Liu L., Du Q., Wu H., Xu H., Sun J. (2020). Loganin and catalpol exert cooperative ameliorating effects on podocyte apoptosis upon diabetic nephropathy by targeting AGEs-RAGE signaling.. Life Sci..

[25] Chen D.Q., Wu X.Q., Chen L., Hu H.H., Wang Y.N., Zhao Y.Y. (2020). Poricoic acid A as a modulator of TPH-1 expression inhibits renal fibrosis *via* modulating protein stability of β-catenin and β-catenin-mediated transcription.. Ther. Adv. Chronic Dis..

[26] Chen D.Q., Wang Y.N., Vaziri N.D., Chen L., Hu H.H., Zhao Y.Y. (2020). Poricoic acid A activates AMPK to attenuate fibroblast activation and abnormal extracellular matrix remodelling in renal fibrosis.. Phytomedicine.

[27] Wang S., Zeng M., Li B., Kan Y., Zhang B., Zheng X., Feng W. (2020). Raw and salt-processed Achyranthes bidentata attenuate LPS-induced acute kidney injury by inhibiting ROS and apoptosis *via* an estrogen-like pathway.. Biomed. Pharmacother..

[28] Wang X., Wang Z.Y., Zheng J.H., Li S. (2021). TCM network pharmacology: A new trend towards combining computational, experimental and clinical approaches.. Chin. J. Nat. Med..

[29] Li X., Miao F., Xin R., Tai Z., Pan H., Huang H., Yu J., Chen Z., Zhu Q. (2023). Combining network pharmacology, molecular docking, molecular dynamics simulation, and experimental verification to examine the efficacy and immunoregulation mechanism of FHB granules on vitiligo.. Front. Immunol..

[30] Wu N., Yuan T., Yin Z., Yuan X., Sun J., Wu Z., Zhang Q., Redshaw C., Yang S., Dai X., Network Pharmacology and Molecular Docking Study of the Chinese Miao Medicine Sidaxue in the Treatment of Rheumatoid Arthritis (2022). Network pharmacology and molecular docking study of the chinese miao medicine sidaxue in the treatment of rheumatoid arthritis.. Drug Des. Devel. Ther..

[31] Pinzi L., Rastelli G. (2019). Molecular docking: Shifting paradigms in drug discovery.. Int. J. Mol. Sci..

[32] Patil V.S., Harish D.R., Vetrivel U., Roy S., Deshpande S.H., Hegde H.V., Hepatitis C. (2022). Hepatitis C virus NS3/4A inhibition and host immunomodulation by tannins from *Terminalia chebula*: A structural perspective.. Molecules.

[33] Cao Y., Yao W., Yang T., Yang M., Liu Z., Luo H., Cao Z., Chang R., Cui Z., Zuo H., Liu B. (2024). Elucidating the mechanisms of Buyang Huanwu Decoction in treating chronic cerebral ischemia: A combined approach using network pharmacology, molecular docking, and *in vivo* validation.. Phytomedicine.

[34] Wishart D.S., Feunang Y.D., Guo A.C., Lo E.J., Marcu A., Grant J.R., Sajed T., Johnson D., Li C., Sayeeda Z., Assempour N., Iynkkaran I., Liu Y., Maciejewski A., Gale N., Wilson A., Chin L., Cummings R., Le D., Pon A., Knox C., Wilson M. (2018). DrugBank 5.0: A major update to the DrugBank database for 2018.. Nucleic Acids Res..

[35] Zhou Y., Zhang Y., Zhao D., Yu X., Shen X., Zhou Y., Wang S., Qiu Y., Chen Y., Zhu F. (2024). TTD: *Therapeutic target database* describing target druggability information.. Nucleic Acids Res..

[36] Amberger J.S., Bocchini C.A., Schiettecatte F., Scott A.F., Hamosh A. (2015). OMIM.org: Online mendelian inheritance in man (OMIM®), an online catalog of human genes and genetic disorders.. Nucleic Acids Res..

[37] Bardou P., Mariette J., Escudié F., Djemiel C., Klopp C. (2014). jvenn: An interactive Venn diagram viewer.. BMC Bioinformatics.

[38] Szklarczyk D., Kirsch R., Koutrouli M., Nastou K., Mehryary F., Hachilif R., Gable A.L., Fang T., Doncheva N.T., Pyysalo S., Bork P., Jensen L.J., von Mering C. (2023). The STRING database in 2023: Protein–protein association networks and functional enrichment analyses for any sequenced genome of interest.. Nucleic Acids Res..

[39] Otasek D., Morris J.H., Bouças J., Pico A.R., Demchak B. (2019). Cytoscape Automation: Empowering workflow-based network analysis.. Genome Biol..

[40] Lu L., Peng J., Wan P., Peng H., Lu J., Xiong G. (2022). Mechanism of *Tripterygium wilfordii Hook.F.- Trichosanthes kirilowii Maxim* decoction in treatment of diabetic kidney disease based on network pharmacology and molecular docking.. Front. Pharmacol..

[41] Zhou Y., Zhou B., Pache L., Chang M., Khodabakhshi A.H., Tanaseichuk O., Benner C., Chanda S.K. (2019). Metascape provides a biologist-oriented resource for the analysis of systems-level datasets.. Nat. Commun..

[42] Eberhardt J., Santos-Martins D., Tillack A.F., Forli S. (2021). AutoDock vina 1.2.0: New docking methods, expanded force field, and python bindings.. J. Chem. Inf. Model..

[43] Páll S., Abraham M.J., Kutzner C., Hess B., Lindahl E. In *tackling exascale software challenges in molecular dynamics simulations with GROMACS*, solving software challenges for exascale.. International Conference on Exascale Applications and Software, EASC 2014,.

[44] Qu X., Zhai B., Liu Y., Chen Y., Xie Z., Wang Q., Wu Y., Liu Z., Chen J., Mei S., Wu J., You Z., Yu Y., Wang Y. (2022). Pyrroloquinoline quinone ameliorates renal fibrosis in diabetic nephropathy by inhibiting the pyroptosis pathway in C57BL/6 mice and human kidney 2 cells.. Biomed. Pharmacother..

[45] Lin T.J., Huang C.C., Lee M.C., Lee Y.P., Huang W.C., Chuang H.L., Wang I.J. (2024). Effects of *Lactobacillus salivarius* ssp. *salicinius* SA-03 supplementation on reversing phthalate-induced asthma in mice.. Nutrients.

[46] Ye J., Li L., Hu Z., Exploring the Molecular Mechanism of Action of Yinchen Wuling Powder for the Treatment of Hyperlipidemia, Using Network Pharmacology, Molecular Docking, and Molecular Dynamics Simulation (2021). Exploring the molecular mechanism of action of yinchen wuling powder for the treatment of hyperlipidemia, using network pharmacology, molecular docking, and molecular dynamics simulation.. BioMed Res. Int..

[47] Han C., Shen Z., Cui T., Ai S., Gao R., Liu Y., Sui G., Hu H., Li W. (2023). Yi-Shen-Hua-Shi granule ameliorates diabetic kidney disease by the “gut-kidney axis”.. J. Ethnopharmacol..

[48] Liu Y., Wang S., Jin G., Gao K., Wang S., Zhang X., Zhou K., Cai Y., Zhou X., Zhao Z. (2023). Network pharmacology-based study on the mechanism of ShenKang injection in diabetic kidney disease through Keap1/Nrf2/Ho-1 signaling pathway.. Phytomedicine.

[49] Ji J., Tao P., Wang Q., Cui M., Cao M., Xu Y. (2023). Emodin attenuates diabetic kidney disease by inhibiting ferroptosis *via* upregulating Nrf2 expression.. Aging (Albany NY).

[50] Yang D., Wang T., Long M., Li P. (2020). Quercetin: Its main pharmacological activity and potential application in clinical medicine.. Oxid. Med. Cell. Longev..

[51] Yang Y., Chen Z., Zhao X., Xie H., Du L., Gao H., Xie C. (2022). Mechanisms of Kaempferol in the treatment of diabetes: A comprehensive and latest review.. Front. Endocrinol..

[52] Sheng H., Zhang D., Zhang J., Zhang Y., Lu Z., Mao W., Liu X., Zhang L. (2022). Kaempferol attenuated diabetic nephropathy by reducing apoptosis and promoting autophagy through AMPK/mTOR pathways.. Front. Med..

[53] Ward M.G., Li G., Barbosa-Lorenzi V.C., Hao M. (2017). Stigmasterol prevents glucolipotoxicity induced defects in glucose-stimulated insulin secretion.. Sci. Rep..

[54] Lei L., Zhao J., Liu X.Q., Chen J., Qi X.M., Xia L.L., Wu Y.G. (2021). Wogonin alleviates kidney tubular epithelial injury in diabetic nephropathy by inhibiting PI3K/Akt/NF-κB signaling pathways.. Drug Des. Devel. Ther..

[55] Babu S., Krishnan M., Rajagopal P., Periyasamy V., Veeraraghavan V., Govindan R., Jayaraman S. (2020). Beta-sitosterol attenuates insulin resistance in adipose tissue *via* IRS-1/Akt mediated insulin signaling in high fat diet and sucrose induced type-2 diabetic rats.. Eur. J. Pharmacol..

[56] Tang X., Yan T., Wang S., Liu Q., Yang Q., Zhang Y., Li Y., Wu Y., Liu S., Ma Y., Yang L. (2024). Treatment with β-sitosterol ameliorates the effects of cerebral ischemia/reperfusion injury by suppressing cholesterol overload, endoplasmic reticulum stress, and apoptosis.. Neural Regen. Res..

[57] Yang M., Kan L., Wu L., Zhu Y., Wang Q. (2019). Effect of baicalin on renal function in patients with diabetic nephropathy and its therapeutic mechanism.. Exp. Ther. Med..

[58] Kuo Y.H., Lin C.H., Shih C.C. (2016). Dehydroeburicoic acid from antrodia camphorata prevents the diabetic and dyslipidemic state *via* modulation of glucose transporter 4, peroxisome proliferator-activated receptor α expression and AMP-activated protein kinase phosphorylation in high-fat-fed mice.. Int. J. Mol. Sci..

[59] Kashani K., Rosner M.H., Ostermann M. (2020). Creatinine: From physiology to clinical application.. Eur. J. Intern. Med..

[60] Huang T., Wu T., Wu Y., Li X., Tan J., Shen C., Xiong S., Feng Z., Gao S., Li H., Cai W. (2023). Long-term statins administration exacerbates diabetic nephropathy *via* ectopic fat deposition in diabetic mice.. Nat. Commun..

[61] Calle P., Hotter G. (2020). Macrophage phenotype and fibrosis in diabetic nephropathy.. Int. J. Mol. Sci..

[62] Rayego-Mateos S., Rodrigues-Diez R.R., Fernandez-Fernandez B., Mora-Fernández C., Marchant V., Donate-Correa J., Navarro-González J.F., Ortiz A., Ruiz-Ortega M. (2023). Targeting inflammation to treat diabetic kidney disease: The road to 2030.. Kidney Int..

[63] Bertheloot D., Latz E., Franklin B.S. (2021). Necroptosis, pyroptosis and apoptosis: An intricate game of cell death.. Cell. Mol. Immunol..

[64] Quarato G., Mari L., Barrows N.J., Yang M., Ruehl S., Chen M.J., Guy C.S., Low J., Chen T., Green D.R. (2023). Mitophagy restricts BAX/BAK-independent, Parkin-mediated apoptosis.. Sci. Adv..

[65] Liu M., Wang W., Gao J., Li F., Shi J., Gong Q. (2020). Icariside II attenuates cerebral ischemia/reperfusion-induced blood–brain barrier dysfunction in rats *via* regulating the balance of MMP9/TIMP1.. Acta Pharmacol. Sin..

[66] Yu Q., Lan T., Ma Z., Wang Z., Zhang C., Jiang Y., Zhao Z. (2023). Lobaplatin induces apoptosis in T24 and 5637 bladder cancer cells by regulating Bcl-2 and Bax expression and inhibiting the PI3K/Akt signaling pathway.. Transl. Androl. Urol..

[67] Holota R., Dečmanová V., Alexovič Matiašová A., Košuth J., Slovinská L., Pačut L., Tomori Z., Daxnerová Z., Ševc J. (2024). Cleaved caspase-3 is present in the majority of glial cells in the intact rat spinal cord during postnatal life.. Histochem. Cell Biol..

[68] Pal R., Bhadada S.K. (2023). AGEs accumulation with vascular complications, glycemic control and metabolic syndrome: A narrative review.. Bone.

[69] Meng F., Zhang Z., Chen C., Liu Y., Yuan D., Hei Z., Luo G. (2021). PI3K/AKT activation attenuates acute kidney injury following liver transplantation by inducing FoxO3a nuclear export and deacetylation.. Life Sci..

[70] Chen Y., Zhang Z., Jin W., Li Z., Bao C., He C., Guo Y., Li C. (2022). Integrative analyses of antler cartilage transcriptome and proteome of gansu red deer (*Cervus elaphus kansuensis*) at different growth stages.. Animals.

[71] Xu Z., Jia K., Wang H., Gao F., Zhao S., Li F., Hao J. (2021). METTL14-regulated PI3K/Akt signaling pathway *via* PTEN affects HDAC5-mediated epithelial–mesenchymal transition of renal tubular cells in diabetic kidney disease.. Cell Death Dis..

[72] Chang C., Huang K., Xu X., Duan R., Yu T., Chu X., Chen C., Li B., Yang T. (2024). MiR-23a-5p alleviates chronic obstructive pulmonary disease through targeted regulation of RAGE-ROS pathway.. Respir. Res..

[73] Bao J.M., He M.Y., Liu Y.W., Lu Y.J., Hong Y.Q., Luo H.H., Ren Z.L., Zhao S.C., Jiang Y. (2015). AGE/RAGE/Akt pathway contributes to prostate cancer cell proliferation by promoting Rb phosphorylation and degradation.. Am. J. Cancer Res..

[74] Sharma I., Tupe R.S., Wallner A.K., Kanwar Y.S. (2018). Contribution of myo-inositol oxygenase in AGE:RAGE-mediated renal tubulointerstitial injury in the context of diabetic nephropathy.. Am. J. Physiol. Renal Physiol..

[75] Ahmad A., Biersack B., Li Y., Kong D., Bao B., Schobert R., Padhye S., Sarkar F. (2013). Targeted regulation of PI3K/Akt/mTOR/NF-κB signaling by indole compounds and their derivatives: Mechanistic details and biological implications for cancer therapy.. Anticancer. Agents Med. Chem..

[76] Kang J., Wang Y., Guo X., He X., Liu W., Chen H., Wang Z., Lin A., Kang X. (2022). N-acetylserotonin protects PC12 cells from hydrogen peroxide induced damage through ROS mediated PI3K / AKT pathway.. Cell Cycle.

