# Supplementary material for: Elucidating the Mechanism of Jisheng Shenqi Pills in the Treatment of Diabetic Kidney Disease: Network Pharmacology Combined with Experimental Verification
Source: Endocr Metab Immune Disord Drug Targets. 2024 Aug 9;25(13):1082–98. doi: 10.2174/0118715303339433240805045749 (PMC12715395; doi:10.2174/0118715303339433240805045749)
Supplement: Supplementary file 1 — Supplementary material is available on the publisher’s website along with the published article. [file EMIDDT-25-13-1082_SD1.pdf]

SUPPLEMENTARY MATERIAL

Elucidating the Mechanism of Jisheng Shenqi Pills in the Treatment of Diabetic Kidney Disease: Network Pharmacology Combined with Experimental Verification

Xiaoshu Ma[1,2] and Guangju Zhou[2,*]

[1]*College of Clinical Medicine, North Sichuan Medical College, Nanchong, China;* [2]*Department of Endocrinology, Affiliated Hospital of North Sichuan Medical College, Nanchong, China*

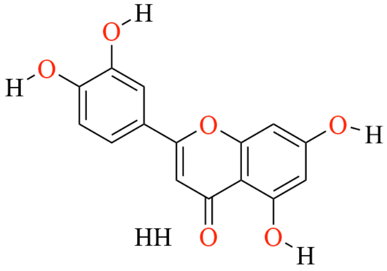

Quercetin

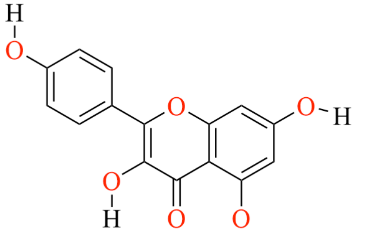

Kaempferol

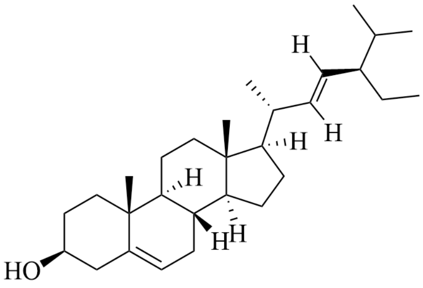

Stigmasterol

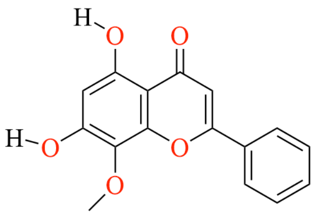

Wogonin

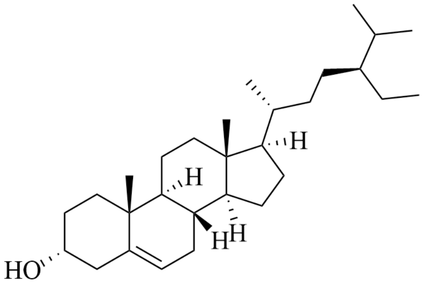

Beta-sitosterol

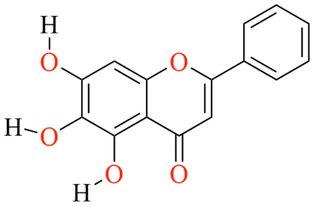

Baicalein

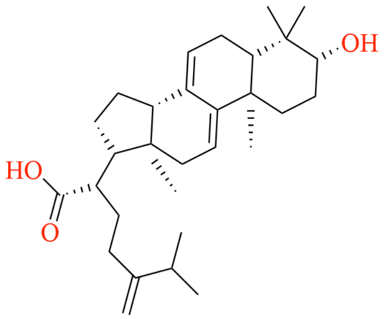

Dehydroeburicoic acid
